# Dibromido{2-(morpholin-4-yl)-*N*-[1-(2-pyrid­yl)ethyl­idene]ethanamine-κ^3^ *N*,*N*′,*N*′′}cadmium

**DOI:** 10.1107/S160053681100554X

**Published:** 2011-02-19

**Authors:** Nura Suleiman Gwaram, Hamid Khaledi, Hapipah Mohd Ali

**Affiliations:** a Department of Chemistry, University of Malaya, 50603 Kuala Lumpur, Malaysia

## Abstract

The Cd^II^ ion in the title compound, [CdBr_2_(C_13_H_19_N_3_O)], is five-coordinated by the *N*,*N*′,*N*′′-tridentate Schiff base ligand and two Br atoms in a distorted square-pyramidal geometry. In the crystal, inter­molecular C—H⋯O and C—H⋯Br hydrogen bonds link adjacent mol­ecules into layers parallel to the *ab* plane. An intra­molecular C—H⋯Br inter­action is also observed.

## Related literature

For the crystal structure of the analogous CdCl_2_ complex, see: Ikmal Hisham *et al.* (2010 ▶). For the crystal structures of similar CdBr_2_ complexes, see: Bermejo *et al.* (1999 ▶, 2003 ▶). For a description of the geometry of complexes with five-coordinate metal atoms, see: Addison *et al.* (1984 ▶).
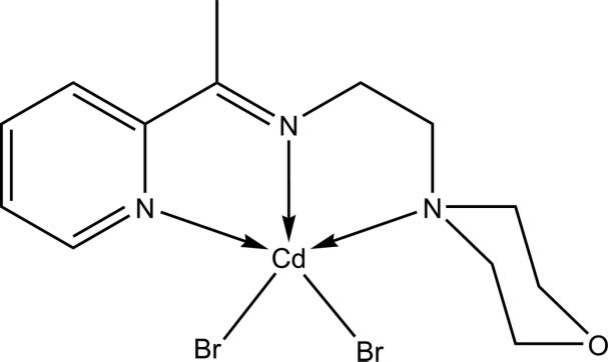


## Experimental

### 

#### Crystal data

[CdBr_2_(C_13_H_19_N_3_O)]
*M* *_r_* = 505.53
Orthorhombic, 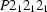

*a* = 9.1906 (8) Å
*b* = 12.2604 (10) Å
*c* = 14.7499 (12) Å
*V* = 1662.0 (2) Å^3^
*Z* = 4
Mo *K*α radiation
μ = 6.12 mm^−1^
*T* = 100 K
0.33 × 0.27 × 0.19 mm

#### Data collection

Bruker APEXII CCD diffractometer
Absorption correction: multi-scan (*SADABS*; Sheldrick, 1996 ▶) *T*_min_ = 0.237, *T*_max_ = 0.389
20236 measured reflections
3642 independent reflections
3445 reflections with *I* > 2σ(*I*)
*R*_int_ = 0.032

#### Refinement

*R*[*F* ^2^ > 2σ(*F* ^2^)] = 0.021
*wR*(*F* ^2^) = 0.041
*S* = 1.09
3642 reflections
182 parameters
H-atom parameters constrained
Δρ_max_ = 0.73 e Å^−3^
Δρ_min_ = −0.54 e Å^−3^
Absolute structure: Flack (1983 ▶), 1556 Friedel pairs
Flack parameter: 0.023 (9)

### 

Data collection: *APEX2* (Bruker, 2007 ▶); cell refinement: *SAINT* (Bruker, 2007 ▶); data reduction: *SAINT*; program(s) used to solve structure: *SHELXS97* (Sheldrick, 2008 ▶); program(s) used to refine structure: *SHELXL97* (Sheldrick, 2008 ▶); molecular graphics: *X-SEED* (Barbour, 2001 ▶); software used to prepare material for publication: *SHELXL97* and *publCIF* (Westrip, 2010 ▶).

## Supplementary Material

Crystal structure: contains datablocks I, global. DOI: 10.1107/S160053681100554X/bg2390sup1.cif

Structure factors: contains datablocks I. DOI: 10.1107/S160053681100554X/bg2390Isup2.hkl

Additional supplementary materials:  crystallographic information; 3D view; checkCIF report

## Figures and Tables

**Table 1:** Hydrogen-bond geometry (Å, °)

*D*—H⋯*A*	*D*—H	H⋯*A*	*D*⋯*A*	*D*—H⋯*A*
C3—H3⋯O1^i^	0.95	2.42	3.132 (4)	131
C7—H7*B*⋯Br1^ii^	0.98	2.92	3.840 (4)	157
C10—H10*A*⋯O1^iii^	0.99	2.45	3.383 (4)	156
C11—H11*B*⋯Br2	0.99	2.91	3.727 (4)	141

Symmetry codes: (i) 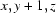; (ii) 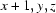; (iii) 
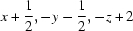
.

**Fig. 1.**
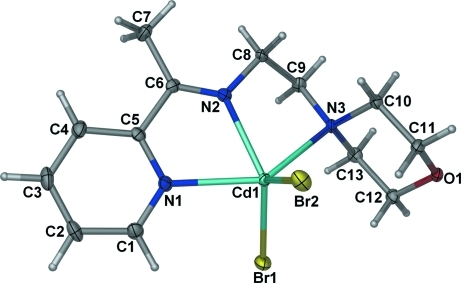
Thermal ellipsoid plot of the title compound at the 50% probability level.

## supplementary crystallographic information

### Comment

The title compound was obtained upon the reaction of 2-morpholino-*N*-[1-(2-pyridyl)ethylidene]ethanamine with Cd^II^ ion in the presence of potassium bromide. Similar to the structure of the analogous CdCl_2_ complex (Ikmal Hisham *et al.*, 2010), the metal center is five-coordinated by the *N,N',N"*-tridentate Schiff base ligand and two halogen atoms. The geometry of the complexes can be determined by using the index τ = (*β*-*α*)/60, where *β* is the largest angle and *α* is the second one around the metal center. For an ideal square-pyramid τ is 0, while it is 1 in a perfect trigonal-bipyramid (Addison *et al.*,1984). The τ value in the present structure is calculated to be 0.18, indicative of a distorted square-pyramidal geometry. The Cd—Br bond lengths in the complex are in agreement with the values reported in the literature (Bermejo *et al.*, 1999; Bermejo *et al.*, 2003). In the crystal, the adjacent molecules are connected together *via* C—H···O and C—H···Br hydrogen bonds, forming infinite layers parallel to the *ab* plane. Moreover an intramolecluar C—H···Br occurs.

### Experimental

A mixture of 2-acetylpyridine (0.20 g, 1.65 mmol) and 4-(2-aminoethyl)morpholine (0.21 g, 1.65 mmol) in ethanol (20 ml) was refluxed. After 2 hr a solution of cadmium(II) acetate dihydrate (0.44 g, 1.65 mmol) and potassium bromide (0.196 g, 1.65 mmol) in a minimum amount of water was added. The resulting solution was refluxed for 30 min, and then left at room temperature. The crystals of the title complex were obtained in a few days.

### Refinement

H atoms were positioned geometrically (C-H: 0.95Å; C-H_2_: 0.99Å;C-H_3_: 0.98Å) and allowed to ride. *U*iso(H) set to 1.2–1.5 *U*eq(C).

### Crystal data

**Table:**

[CdBr_2_(C_13_H_19_N_3_O)]	*F*(000) = 976
*M**_r_* = 505.53	*D*_x_ = 2.020 Mg m^−^^3^
Orthorhombic, *P*2_1_2_1_2_1_	Mo *K*α radiation, λ = 0.71073 Å
Hall symbol: P 2ac 2ab	Cell parameters from 5963 reflections
*a* = 9.1906 (8) Å	θ = 2.6–30.7°
*b* = 12.2604 (10) Å	µ = 6.12 mm^−^^1^
*c* = 14.7499 (12) Å	*T* = 100 K
*V* = 1662.0 (2) Å^3^	Block, colorless
*Z* = 4	0.33 × 0.27 × 0.19 mm

### Data collection

**Table:**

Bruker APEXII CCD diffractometer	3642 independent reflections
Radiation source: fine-focus sealed tube	3445 reflections with *I* > 2σ(*I*)
graphite	*R*_int_ = 0.032
φ and ω scans	θ_max_ = 27.0°, θ_min_ = 2.2°
Absorption correction: multi-scan (*SADABS*; Sheldrick, 1996)	*h* = −11→11
*T*_min_ = 0.237, *T*_max_ = 0.389	*k* = −15→15
20236 measured reflections	*l* = −18→18

### Refinement

**Table:**

Refinement on *F*^2^	Secondary atom site location: difference Fourier map
Least-squares matrix: full	Hydrogen site location: inferred from neighbouring sites
*R*[*F*^2^ > 2σ(*F*^2^)] = 0.021	H-atom parameters constrained
*wR*(*F*^2^) = 0.041	*w* = 1/[σ^2^(*F*_o_^2^) + (0.006*P*)^2^ + 1.6071*P*] where *P* = (*F*_o_^2^ + 2*F*_c_^2^)/3
*S* = 1.09	(Δ/σ)_max_ = 0.001
3642 reflections	Δρ_max_ = 0.73 e Å^−^^3^
182 parameters	Δρ_min_ = −0.54 e Å^−^^3^
0 restraints	Absolute structure: Flack (1983), 1556 Friedel pairs
Primary atom site location: structure-invariant direct methods	Flack parameter: 0.023 (9)

### Special details

**Table:**

Geometry. All e.s.d.'s (except the e.s.d. in the dihedral angle between two l.s. planes) are estimated using the full covariance matrix. The cell e.s.d.'s are taken into account individually in the estimation of e.s.d.'s in distances, angles and torsion angles; correlations between e.s.d.'s in cell parameters are only used when they are defined by crystal symmetry. An approximate (isotropic) treatment of cell e.s.d.'s is used for estimating e.s.d.'s involving l.s. planes.
Refinement. Refinement of *F*^2^ against ALL reflections. The weighted *R*-factor *wR* and goodness of fit *S* are based on *F*^2^, conventional *R*-factors *R* are based on *F*, with *F* set to zero for negative *F*^2^. The threshold expression of *F*^2^ > σ(*F*^2^) is used only for calculating *R*-factors(gt) *etc*. and is not relevant to the choice of reflections for refinement. *R*-factors based on *F*^2^ are statistically about twice as large as those based on *F*, and *R*- factors based on ALL data will be even larger.

### Fractional atomic coordinates and isotropic or equivalent isotropic displacement parameters (Å^2^)

**Table:**

	*x*	*y*	*z*	*U*_iso_*/*U*_eq_	
Cd1	0.03808 (2)	0.00442 (2)	0.919266 (13)	0.01367 (5)	
Br1	−0.21463 (3)	0.00329 (4)	0.84484 (2)	0.02368 (7)	
Br2	0.07480 (3)	−0.00774 (4)	1.092730 (19)	0.02169 (7)	
O1	−0.0135 (3)	−0.34931 (19)	0.93888 (17)	0.0151 (6)	
N1	0.0632 (3)	0.1977 (2)	0.90768 (19)	0.0163 (6)	
N2	0.2634 (3)	0.0505 (2)	0.86157 (18)	0.0143 (6)	
N3	0.1405 (3)	−0.1675 (2)	0.85991 (18)	0.0127 (6)	
C1	−0.0399 (4)	0.2704 (3)	0.9296 (2)	0.0194 (7)	
H1	−0.1334	0.2443	0.9463	0.023*	
C2	−0.0157 (5)	0.3826 (3)	0.9291 (3)	0.0225 (9)	
H2	−0.0908	0.4322	0.9451	0.027*	
C3	0.1197 (4)	0.4192 (3)	0.9049 (3)	0.0240 (8)	
H3	0.1400	0.4951	0.9051	0.029*	
C4	0.2272 (4)	0.3455 (3)	0.8800 (2)	0.0209 (8)	
H4	0.3206	0.3701	0.8616	0.025*	
C5	0.1948 (4)	0.2342 (3)	0.8827 (2)	0.0145 (7)	
C6	0.3044 (4)	0.1495 (3)	0.8549 (2)	0.0146 (7)	
C7	0.4496 (4)	0.1874 (3)	0.8209 (3)	0.0253 (8)	
H7A	0.4380	0.2199	0.7606	0.038*	
H7B	0.5162	0.1252	0.8172	0.038*	
H7C	0.4894	0.2420	0.8627	0.038*	
C8	0.3532 (4)	−0.0426 (2)	0.8376 (2)	0.0164 (7)	
H8A	0.4034	−0.0709	0.8922	0.020*	
H8B	0.4278	−0.0207	0.7928	0.020*	
C9	0.2553 (4)	−0.1303 (3)	0.7977 (2)	0.0176 (7)	
H9A	0.2093	−0.1016	0.7419	0.021*	
H9B	0.3160	−0.1937	0.7802	0.021*	
C10	0.2041 (4)	−0.2361 (3)	0.9323 (2)	0.0167 (7)	
H10A	0.2692	−0.1912	0.9705	0.020*	
H10B	0.2630	−0.2950	0.9047	0.020*	
C11	0.0853 (4)	−0.2858 (3)	0.9907 (2)	0.0181 (7)	
H11A	0.1303	−0.3323	1.0378	0.022*	
H11B	0.0313	−0.2267	1.0217	0.022*	
C12	−0.0787 (4)	−0.2837 (3)	0.8695 (2)	0.0177 (7)	
H12A	−0.1356	−0.2242	0.8978	0.021*	
H12B	−0.1463	−0.3290	0.8333	0.021*	
C13	0.0361 (4)	−0.2354 (3)	0.8079 (2)	0.0174 (7)	
H13A	0.0894	−0.2950	0.7770	0.021*	
H13B	−0.0114	−0.1901	0.7609	0.021*	

### Atomic displacement parameters (Å^2^)

**Table:**

	*U*^11^	*U*^22^	*U*^33^	*U*^12^	*U*^13^	*U*^23^
Cd1	0.01446 (9)	0.01227 (9)	0.01429 (9)	0.00005 (13)	0.00293 (7)	0.00048 (13)
Br1	0.01711 (14)	0.02244 (15)	0.03150 (16)	0.0017 (2)	−0.00424 (12)	0.0024 (2)
Br2	0.02749 (16)	0.02287 (17)	0.01472 (13)	0.00513 (19)	−0.00019 (11)	−0.00188 (19)
O1	0.0147 (14)	0.0118 (11)	0.0190 (14)	−0.0018 (10)	−0.0005 (10)	0.0025 (10)
N1	0.0184 (15)	0.0152 (13)	0.0154 (14)	0.0012 (11)	0.0020 (12)	0.0039 (11)
N2	0.0147 (15)	0.0163 (13)	0.0120 (13)	0.0018 (11)	0.0003 (11)	0.0003 (11)
N3	0.0155 (14)	0.0100 (13)	0.0128 (14)	−0.0002 (11)	0.0015 (11)	0.0010 (11)
C1	0.0217 (18)	0.0191 (16)	0.0173 (18)	0.0019 (15)	0.0056 (15)	0.0012 (14)
C2	0.032 (2)	0.0147 (16)	0.021 (2)	0.0080 (16)	−0.0011 (17)	0.0001 (15)
C3	0.034 (2)	0.0116 (15)	0.027 (2)	−0.0018 (14)	−0.0087 (17)	−0.0008 (15)
C4	0.0203 (18)	0.0163 (17)	0.0260 (19)	−0.0043 (14)	−0.0043 (15)	0.0000 (14)
C5	0.0152 (18)	0.0154 (16)	0.0128 (17)	0.0028 (13)	−0.0030 (15)	0.0017 (14)
C6	0.0145 (17)	0.0176 (16)	0.0116 (16)	−0.0009 (13)	−0.0011 (13)	0.0041 (13)
C7	0.0204 (19)	0.0187 (17)	0.037 (2)	−0.0055 (15)	0.0066 (17)	0.0005 (15)
C8	0.0134 (17)	0.0135 (15)	0.0224 (18)	0.0018 (12)	0.0040 (14)	0.0031 (13)
C9	0.0202 (18)	0.0164 (17)	0.0162 (17)	0.0017 (13)	0.0064 (14)	−0.0013 (14)
C10	0.0169 (17)	0.0155 (16)	0.0178 (18)	0.0023 (13)	−0.0007 (15)	0.0045 (14)
C11	0.0149 (17)	0.0172 (17)	0.0223 (18)	−0.0020 (13)	−0.0011 (14)	0.0052 (14)
C12	0.0183 (18)	0.0150 (16)	0.0198 (18)	−0.0016 (13)	−0.0013 (15)	−0.0005 (13)
C13	0.0210 (18)	0.0165 (16)	0.0147 (16)	0.0011 (14)	−0.0007 (14)	−0.0049 (13)

### Geometric parameters (Å, °)

**Table:**

Cd1—N2	2.309 (3)	C4—H4	0.9500
Cd1—N1	2.388 (3)	C5—C6	1.504 (5)
Cd1—N3	2.469 (3)	C6—C7	1.500 (5)
Cd1—Br1	2.5690 (4)	C7—H7A	0.9800
Cd1—Br2	2.5851 (4)	C7—H7B	0.9800
O1—C11	1.420 (4)	C7—H7C	0.9800
O1—C12	1.433 (4)	C8—C9	1.520 (5)
N1—C1	1.340 (4)	C8—H8A	0.9900
N1—C5	1.341 (4)	C8—H8B	0.9900
N2—C6	1.275 (4)	C9—H9A	0.9900
N2—C8	1.452 (4)	C9—H9B	0.9900
N3—C9	1.471 (4)	C10—C11	1.518 (5)
N3—C10	1.479 (4)	C10—H10A	0.9900
N3—C13	1.484 (4)	C10—H10B	0.9900
C1—C2	1.393 (5)	C11—H11A	0.9900
C1—H1	0.9500	C11—H11B	0.9900
C2—C3	1.370 (6)	C12—C13	1.514 (5)
C2—H2	0.9500	C12—H12A	0.9900
C3—C4	1.388 (5)	C12—H12B	0.9900
C3—H3	0.9500	C13—H13A	0.9900
C4—C5	1.397 (5)	C13—H13B	0.9900
			
N2—Cd1—N1	69.15 (9)	C6—C7—H7B	109.5
N2—Cd1—N3	74.71 (9)	H7A—C7—H7B	109.5
N1—Cd1—N3	141.76 (9)	C6—C7—H7C	109.5
N2—Cd1—Br1	130.89 (7)	H7A—C7—H7C	109.5
N1—Cd1—Br1	93.56 (7)	H7B—C7—H7C	109.5
N3—Cd1—Br1	100.87 (6)	N2—C8—C9	108.3 (3)
N2—Cd1—Br2	105.18 (7)	N2—C8—H8A	110.0
N1—Cd1—Br2	96.63 (7)	C9—C8—H8A	110.0
N3—Cd1—Br2	104.59 (6)	N2—C8—H8B	110.0
Br1—Cd1—Br2	122.729 (12)	C9—C8—H8B	110.0
C11—O1—C12	110.1 (2)	H8A—C8—H8B	108.4
C1—N1—C5	118.8 (3)	N3—C9—C8	113.7 (3)
C1—N1—Cd1	125.1 (2)	N3—C9—H9A	108.8
C5—N1—Cd1	116.0 (2)	C8—C9—H9A	108.8
C6—N2—C8	124.2 (3)	N3—C9—H9B	108.8
C6—N2—Cd1	121.8 (2)	C8—C9—H9B	108.8
C8—N2—Cd1	114.02 (19)	H9A—C9—H9B	107.7
C9—N3—C10	110.1 (3)	N3—C10—C11	110.7 (3)
C9—N3—C13	108.4 (3)	N3—C10—H10A	109.5
C10—N3—C13	108.1 (2)	C11—C10—H10A	109.5
C9—N3—Cd1	103.29 (18)	N3—C10—H10B	109.5
C10—N3—Cd1	112.3 (2)	C11—C10—H10B	109.5
C13—N3—Cd1	114.57 (19)	H10A—C10—H10B	108.1
N1—C1—C2	122.8 (3)	O1—C11—C10	112.0 (3)
N1—C1—H1	118.6	O1—C11—H11A	109.2
C2—C1—H1	118.6	C10—C11—H11A	109.2
C3—C2—C1	118.0 (4)	O1—C11—H11B	109.2
C3—C2—H2	121.0	C10—C11—H11B	109.2
C1—C2—H2	121.0	H11A—C11—H11B	107.9
C2—C3—C4	120.1 (3)	O1—C12—C13	110.9 (3)
C2—C3—H3	119.9	O1—C12—H12A	109.5
C4—C3—H3	119.9	C13—C12—H12A	109.5
C3—C4—C5	118.4 (3)	O1—C12—H12B	109.5
C3—C4—H4	120.8	C13—C12—H12B	109.5
C5—C4—H4	120.8	H12A—C12—H12B	108.0
N1—C5—C4	121.7 (3)	N3—C13—C12	111.1 (3)
N1—C5—C6	116.6 (3)	N3—C13—H13A	109.4
C4—C5—C6	121.6 (3)	C12—C13—H13A	109.4
N2—C6—C7	125.7 (3)	N3—C13—H13B	109.4
N2—C6—C5	116.0 (3)	C12—C13—H13B	109.4
C7—C6—C5	118.2 (3)	H13A—C13—H13B	108.0
C6—C7—H7A	109.5		

### Hydrogen-bond geometry (Å, °)

**Table:**

*D*—H···*A*	*D*—H	H···*A*	*D*···*A*	*D*—H···*A*
C3—H3···O1^i^	0.95	2.42	3.132 (4)	131
C7—H7B···Br1^ii^	0.98	2.92	3.840 (4)	157
C10—H10A···O1^iii^	0.99	2.45	3.383 (4)	156
C11—H11B···Br2	0.99	2.91	3.727 (4)	141

Symmetry codes: (i) *x*, *y*+1, *z*; (ii) *x*+1, *y*, *z*; (iii) *x*+1/2, −*y*−1/2, −*z*+2.
